# 

**Published:** 1890-01-18

**Authors:** 


PRICE ONE PENNY.

No. 178, Vol. VII.—January 18th, 1890.

[Registered at the General Post Office as a Newspaper for transmission in the United Kingdom and Abroad.

WITH EXTRA NURSING SUPPLEMENT.

Per Annum, 6s. 6d., post free.

Monthly Parts, 6d.; post free 7½d.

Ditto per Annum, 7s. 6d., post free.

